# Mid-upper arm circumference predicts death in adult patients admitted to a TB ward in the Philippines: A prospective cohort study

**DOI:** 10.1371/journal.pone.0218193

**Published:** 2019-06-27

**Authors:** Nathaniel Lee, Laura V. White, Flora P. Marin, Naomi R. Saludar, Marietta B. Solante, Rosario J. C. Tactacan-Abrenica, Rugaiya W. Calapis, Motoi Suzuki, Nobuo Saito, Koya Ariyoshi, Christopher M. Parry, Tansy Edwards, Sharon E. Cox

**Affiliations:** 1 School of Tropical Medicine and Global Health, Nagasaki University, Nagasaki, Japan; 2 Royal Free Hospital, London, United Kingdom; 3 San Lazaro Hospital, Manila, the Philippines; 4 The Lung Center, Manila, the Philippines; 5 San Lazaro Hospital PMDT Treatment Center, Manila, the Philippines; 6 Institute of Tropical Medicine, Nagasaki University, Nagasaki, Japan; 7 Institute of Infection and Global Health, University of Liverpool, Liverpool, United Kingdom; 8 Tropical Epidemiology Group, London School of Hygiene and Tropical Medicine, London, United Kingdom; 9 Faculty of Population Health, London School of Hygiene and Tropical Medicine, London, United Kingdom; Universidade Federal de Goias, BRAZIL

## Abstract

**Background:**

The Philippines is ranked 3^rd^ globally for tuberculosis incidence (554/100,000 population). The tuberculosis ward at San Lazaro Hospital, Manila receives 1,800–2,000 admissions of acutely unwell patients per year with high mortality. Objectives of this prospective cohort study were to quantify the association of under-nutrition (primary) and diabetes (secondary) with inpatient mortality occurring between 3–28 days of hospital admission in patients with suspected or previously diagnosed TB.

**Methods and results:**

We enrolled 360 adults (≥18 years); 348 were eligible for the primary analysis (alive on day 3). Clinical, laboratory, anthropometric and enhanced tuberculosis diagnostic data were collected at admission with telephone tracing for mortality up to 6 months post-discharge. In the primary analysis population (mean age 45 years, SD = 15.0 years, 70% male), 58 (16.7%) deaths occurred between day 3–28 of admission; 70 (20.1%) between day 3 and discharge and documented total post-day 3 mortality including follow-up was 96 (27.6%). In those in whom it could be assessed, body mass index (BMI) ranged from 11.2–30.6 kg/m^2^ and 141/303 (46.5%) had moderate/severe undernutrition (BMI<17 kg/m^2^). A sex-specific cut-off for mid-upper arm circumference predictive of BMI<17 kg/m^2^ was associated with inpatient Day 3–28 mortality in males (AOR = 5.04, 95% CI: 1.50–16.86; p = 0.009; p = 0.032 for interaction by sex). The inability to stand for weight/height for BMI assessment was also associated with mortality (AOR = 5.59; 95% CI 2.25–13.89; p<0.001) as was severe compared to normal/mild anaemia (AOR = 9.67; 95% CI 2.48–37.76; p<0.001). No TB specific variables were associated with Day 3–28 mortality, nor was diabetes (HbA1c ≥6.5% or diabetes treatment). Similar effects were observed when the same multivariable model was applied to confirmed TB patients only and to the outcome of all post-day 3 in-patient mortality.

**Conclusion:**

This research supports the use of mid-upper arm circumference for triaging acutely unwell patients and the design and testing of nutrition-based interventions to improve patient outcomes.

## Introduction

Tuberculosis (TB) is the leading cause of death from a single infectious agent [1]. Malnutrition, in this case “under-nutrition” is both a risk factor for and complication of active TB disease [2–4]. Clinical wasting and under-nutrition are common clinical findings in patients infected *with Mycobacterium tuberculosis* (MTB) and are associated with mortality and adverse outcomes [5–7]. A recent systematic review [8] identified under-nutrition as a consistently demonstrable risk factor for death in TB patients on treatment, both within 2 months of treatment initiation as well as late deaths after completion of treatment.

The contribution of under-nutrition to poorer TB treatment outcomes may be mediated through effects on immunity, or altered treatment pharmacokinetics such as increased drug toxicity [9] or decreased drug absorption [10–12]. Both humoral and cell mediated immune responses active in TB disease are negatively affected by under-nutrition, particularly protein energy under-nutrition [13,14]. Immune responses may be important not only against the TB organisms but also against co-infecting organisms [15], which have been associated with increased mortality. In acutely unwell patients, under-nutrition may also increase the risk of inpatient death due to increased risk of severe metabolic and electrolyte disturbances [16–18]. Type 2 Diabetes Mellitus (T2DM) is another known risk factor for active TB [19]. T2DM, especially when poorly controlled, may increase the risk of death and relapse during TB disease [20] whilst TB may also negatively affect glycaemic control [21].

Body Mass Index (BMI; kg/m^2^) is a widely accepted measure to detect under- and over-nutrition, reflecting relative thinness or fatness, but may be difficult to assess in acutely unwell patients, whilst weight is affected by hydration and fluid balance, even when these are not clinically detectable and can affect BMI classification [22]. Mid-upper arm circumference (MUAC) is simple, less affected by fluid balance and is commonly used in young children [22,23] but there are no accepted cut-offs for adults and little data to support such for more severe levels of under-nutrition, which has limited its use [24]. Studies specifically investigating suitable anthropometric measurements in acutely unwell patients with TB are lacking.

The Philippines has a population of around 100 million and is a high burden country for both TB and multidrug resistant TB (MDR-TB), with a recent estimated TB incidence of 554/100,000 population, placing it third in the world for TB incidence [1]. In 2013, the national prevalence of undernutrition (BMI<18.5kg/m^2^) for adults 20 years and older was 9.4% for males and 10.5% for females [25]. The national prevalence of T2DM in 2013 ranged from 4.1–12.8% depending on the criteria used [25].

There is a lack of quality evidence to inform a comprehensive nutritional management strategy for malnourished TB patients [26,27]. The aim of this prospective cohort study was to quantify the association of under-nutrition with inpatient mortality within 28 days of hospital admission of Filipino patients with suspected or previously diagnosed TB. The specific primary objective was to assess the effect of moderate or severe under-nutrition measured as BMI<17 kg/m^2^). A secondary objective was to investigate the impact of diabetes on in-patient mortality.

## Materials and methods

### Study design and setting

This was a prospective cohort study in San Lazaro Hospital (SLH), a single large urban public hospital and tertiary level infectious diseases referral center serving a poor population in Metro Manila in the Philippines with high admission rates to its TB ward [15,28]. Participants were enrolled from July 25^th^, 2016 –May 3^rd^, 2017 and followed up until death or the patient leaving the ward alive (including discharges, transfers or absconding from the ward). The last study participant was discharged on the 8^th^ June 2017. Telephone tracing was conducted at one, two and six months post-discharge to determine vital status and TB treatment status.

### Study participants

Participants were enrolled sequentially from patients with suspected or previously diagnosed TB admitted to the TB ward at SLH. Study nurses checked the ward admissions register twice daily (on weekdays) and approached newly admitted patients for consent to participate within 24 hours of admission. The only exclusion criteria were those aged less than 18 years or if too unwell to give written consent or participate (e.g. unconscious, incoherent, severe shortness of breath or life threatening presentations).

### Primary outcome

The primary outcome was defined as all-cause in-patient mortality between day three and day 28 (D3 and D28) after admission, inclusive (alive or died). Patients transferred or discharged out of the TB ward prior to D28 or still alive on D28 were included as alive.

### Data collection and clinical procedures

At admission, study questionnaires included participant demographics, previous TB symptoms, past medical history, recent food intake and history of weight loss. Research nurses completed anthropometric measurements within 24 hours of admission as previously described [29]. In brief, these included weight (to the nearest 0.1kg; Seca 803 Clara Digitial Personal Non-Medical Scale), height (to nearest 0.5cm; Seca 216 Mechanical Stadiometer), mid-upper arm circumference (MUAC; to the nearest 0.5cm; Seca 201 measuring tape), four-site skinfold measurements (mm units; Harpenden calipers model 68875, Country Technology) and handgrip strength (kg; Jamar Hydraulic Hand Dynamometer Lafayette Instruments, USA). BMI was calculated as kg/m^2^. Our primary exposure was moderate/severe undernutrition defined as BMI <17 kg/m^2^, based upon the WHO cutoff for moderate thinness [30,31]. The MUAC measurement from the non-dominant hand was used in analyses. Moderate/severe under-nutrition using MUAC was defined as ≤18.5 cm for females and ≤20.5 cm for males, following an investigation of the performance of MUAC to predict BMI<17 kg/m^2^ in the same study population [29]. Body fat percentage was calculated using the Durnin and Wormersley equation [32]. Grip strength was analyzed as the maximum recorded value for either hand.

Routine laboratory and clinical data obtained by the hospital was extracted from patient charts. HIV screening (where additional consent was provided) was conducted using standard hospital procedures and Standard Diagnostics Bioline HIV-1/2 Ag/Ab Combo Rapid Test kits. A previous HIV diagnosis or reactive HIV screening was classified as HIV positive. Individuals with a non-reactive screening test were classified as HIV negative. Individuals not agreeing to undergo testing were classified as HIV status unknown. Sputum collection pots and explanations on expectoration were provided to all participants at enrollment and research nurses assisted patients to expectorate and collect samples in the early morning. Sputa samples were assessed using direct sputum smear microscopy using the Ziehl Neelsen stain (AFB; Acid Fast Bacilli smear) and GeneXpert MTB/RIF (Cepheid) testing on all samples. Digital photos of admission chest x-rays (as available) were uploaded to the study database and were assessed by two independent, trained study physicians for results consistent with pulmonary TB and a severity score generated [33]. Venous blood was collected for study-specific assessments of glycated haemoglobin (HbA1c) and C-reactive protein (CRP) (Alere Afinion AS100 point of care analyzer) and additional electrolytes; phosphate, magnesium and calcium, assessed at an external quality assured laboratory (Hi-Precision Diagnostics, Manila). Standard electrolytes, liver and kidney function tests were conducted as per hospital protocols. Critical levels of electrolytes were defined according to published criteria [34] as a corrected calcium <1.5 or >3.4 mmol/L, magnesium <0.3 or >3.3 mmol/L, a phosphate level <0.3 or >1.6 mmol/L, potassium <2.5 or >7 mmol/L, and sodium <120 or >160 mmol/L. Serum calcium was corrected for albumin using the formula “*Corrected calcium = (uncorrected Calcium [mmol/L] + (0*.02*albumin [g/L]) if albumin <40.*0 g/L*” [35]. Where albumin was missing, the average value was used for the purpose of calcium correction. T2DM was defined as HbA1c ≥6.5% or on current diabetes medication. Haemoglobin values at admission were used to generate anaemia classifications using standard WHO cutoffs to define mild, moderate and severe [36]. Systemic Inflammatory Response Syndrome (SIRS) was defined as presence of two or more of the following; body temperature >38.3° or <36.0°C, heart rate > 90 beats per minute, respiratory rate > 20 breaths per minute, and a total white blood cell count <4 or >12 x 10^9^ [37].

A hospital diagnosis of TB was defined as the final diagnosis of the treating clinician extracted from the clinical notes. Study-defined TB included i) any patient with AFB positive sputum smear or MTB detected by GeneXpert, except individuals with high grade positive AFB and no MTB detected by GeneXpert (deemed to be non-TB mycobacterium); ii) receiving anti-TB treatment prior to admission, regardless of bacteriological confirmation; iii) a new chest X-ray indicative of TB, with reported cough of duration ≥2 weeks.

### Sample size

We pre-specified a sample size of 348 patients who survive the first 48 hours after admission (alive on D3) to detect an increase from 15% to 30% mortality between exposure groups, with 5% significance and at least 90% power if the proportion of patients exposed (BMI<17.0 kg/m^2^) was between 30–60% [28].

### Statistical analysis

All data were collected on electronic questionnaires using Open Data Kit (opendatakit.org) software on Android tablets, uploaded to a secure server at the end of every day [38]. Data were analyzed with Stata version 14 (College Station TX; StataCorp LP). The primary analysis population comprised patients still alive on D3. Data summaries were calculated for those that did not survive beyond D3 and for patients who survived beyond D3, with and without BMI values, and combined. Characteristics of patients at the time of admission were summarized as per data type. Univariable associations with in-patient mortality were analysed using logistic regression. MUAC measurement was expected to be more complete than BMI, given that MUAC can be measured in patients that cannot be weighed or have height measured. In an exploratory analysis, the predicted probability of death within D3-D28 was visualized by plotting predicted values of MUAC from unadjusted fractional polynomial logistic regression models, by sex.

The primary analysis approach was to build a multivariable model to demonstrate the effect of under-nutrition (the primary exposure of interest), adjusting for confounders and effect modifiers of the effect of under-nutrition and independent risk factors of in-patient mortality. Under-nutrition was analysed based on two definitions; i) using a categorical variable based on BMI (<17.0, ≥17.0 and missing, due to the numbers of missing data due to patient incapacity) and ii) a binary variable based on MUAC<18.5 cm in females and<20.5 cm in male patients. To build a final multivariable model, age was included *a priori*, and then the important nutrition and anthropometric variables were identified. Inclusion of each additional variable in turn was evaluated by comparing adjusted models with and without the new variable using a likelihood ratio test (LRT), with retention in the adjusted model if the LRT p-value ≤ 0.1. After adjustment for age and important anthropometric variables, variables associated with in-patient mortality (LRT p ≤ 0.1 in univariable analysis) and variables demonstrating a confounding effect on the association between under-nutrition and mortality were investigated in turn with the same retention criterion. Interactions in the final model were also tested and retained based on LRT results.

The final multivariable model was re-fitted in two subsets; study-defined TB patients and bacteriologically confirmed TB patients. After adjustment for the same fixed terms additional clinical factors of interest relevant only to these subgroups were investigated for associations with in-patient mortality.

### Ethical statement

The study protocol and informed consent forms received approval by the Institutional Review Boards of Nagasaki University (NEKKEN) and San Lazaro Hospital. Research nurses obtained informed written consent, and all research nurses completed Good Clinical Practice certification prior to patient enrollment.

## Results and discussion

**Fig 1:**
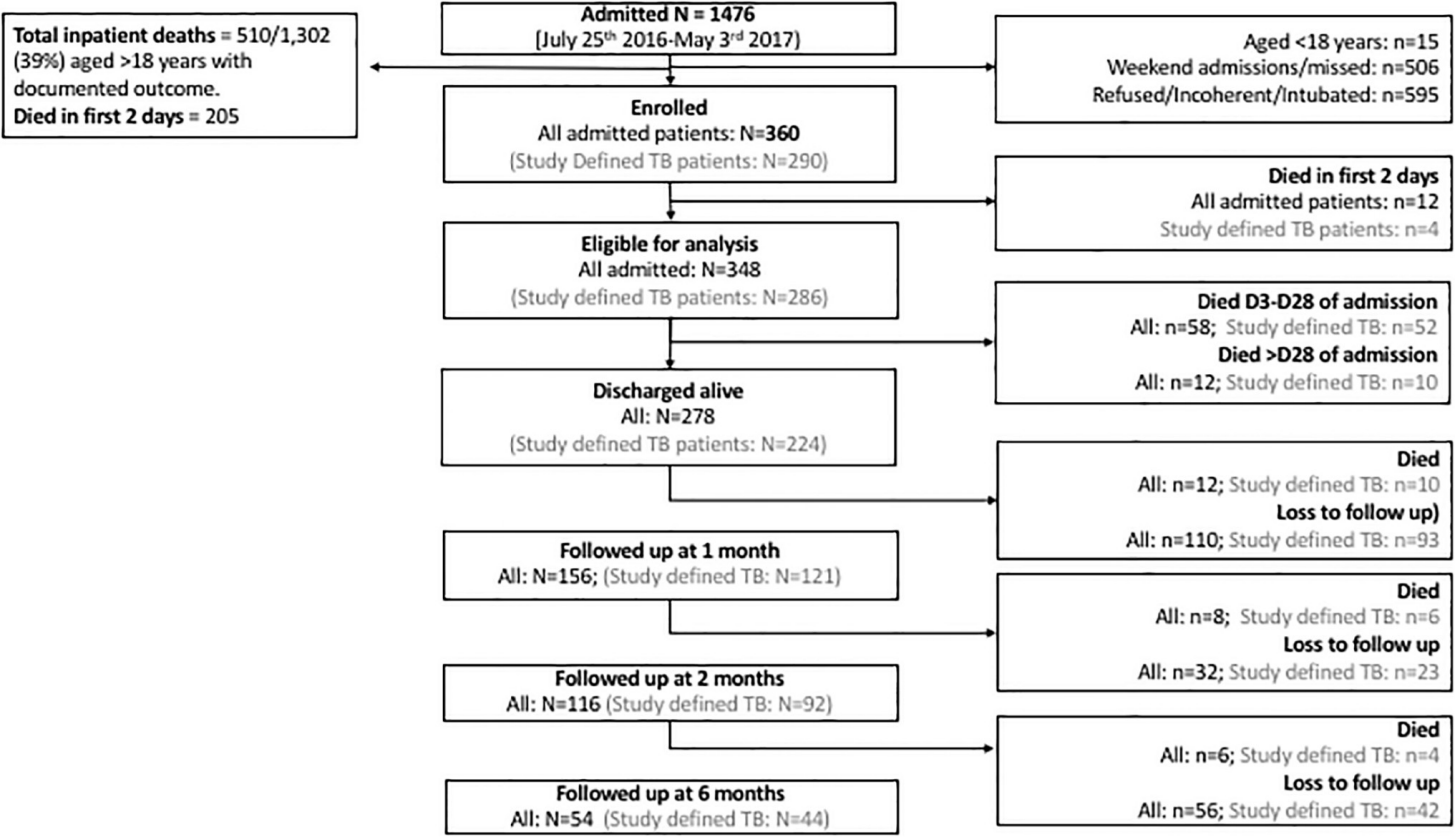
Study flow diagram.

We enrolled 360 individuals from a total of 1476 admissions with known or suspected TB (Fig 1). The main reasons for exclusion were weekend (Friday night to Saturday) or holiday admissions (34.3%), or the patient being too unwell to participate, give consent or refused (40.3%).

### Study participants

Patient characteristics and missing data summaries are presented in Table 1 and S1 Table. In the primary analysis population, 70% were male with a mean age of 45 years (SD 15). Within this group, BMI could not be assessed for 45 patients (12.9%) due to incapacity to stand. Of the 303/348 patients in the primary analysis population with BMI assessed, BMI ranged from 11.2–30.6; 13.5% had moderate undernutrition (BMI 16.0–16.9 kg/m^2^) and 33% severe undernutrition (BMI <16 kg/m^2^). According to our study-defined, sex-specific MUAC cut-offs, 53% of 348 patients had moderate or severe undernutrition. In patients in whom BMI could not be assessed, MUAC was significantly lower than in those with BMI available (p = 0.0001) with more severe anaemia (p = 0.003 test for trend) and a greater probability of one or more critically low electrolytes (p<0.0001). Thus, exclusion of more unwell patients with missing BMI would introduce selection bias.

**Table 1:** Characteristics of study participants.

		Died <D3	Alive after D3 of admission		
Characteristic		N = 12	All (N = 348)	With BMI (N = 303)	BMI missing (N = 45)
Age (years)	mean (SD)	47.8 (18.0)	45.3 (15.0)	45.5 (14.8)	44.0 (16.5)
Age (category)	18–40	5 (41.7)	139 (39.9)	118 (38.9)	21 (46.7)
	41–65	4 (33.3)	177 (50.9)	157 (51.8)	20 (44.4)
	>65	3 (25.0)	32 (9.2)	28 (9.2)	4 (8.9)
Sex	Female	3 (25.0)	106 (30.5)	92 (30.4)	14 (31.1)
	Male	9 (75.0)	242 (69.5)	211 (69.6)	31 (68.9)
Highest Education Achieved	Primary	6 (50.0)	117 (33.6)	99 (32.7)	18 (40.0)
	Secondary	4 (33.3)	169 (48.6)	148 (48.8)	21 (46.7)
	Tertiary	2 (16.7)	48 (13.8)	43 (14.2)	5 (11.1)
	Vocational		14 (4.0)	13 (4.3)	1 (2.2)
Occupation	Not Working	5 (41.7)	152 (44.1)	127 (42.3)	25 (55.6)
	Service and Sales	3 (25.0)	107 (31.0)	97 (32.3)	10 (22.2)
	Trades and Manual Labour	3 (25.0)	63 (18.3)	54 (18.0)	9 (20.0)
	Office Work	1 (8.3)	23 (6.7)	22 (7.3)	1 (2.2)
BMI (kg/m^2^)	Mean (SD)	14.1 (3.1) [N = 5]	-	17.9 (3.7)	-
BMI (category; kg/m^2^)	> = 17kg/m2	-	-	162 (53.5)	
	<17kg/m2	-	-	141 (46.5)	
MUAC (cm)	Mean (SD)	18.1 (3.3)	20.0 (3.6)	20.3 (3.5)	18.0 (3.7)^a^
Any food intake in the past 24 hours	No		14 (6.1)	11 (5.2)	3 (17.6)
	Yes	4 (100.0)	214 (93.9)	200 (94.8)	14 (82.4)
Food intake over past month	No decrease		99 (43.4)	93 (44.1)	6 (35.3)
	Moderate decrease	3 (75.0)	112 (49.1)	104 (49.3)	8 (47.1)
	Severe decrease	1 (25.0)	17 (7.5)	14 (6.6)	3 (17.6)
Diabetic	Non-diabetic (HbA1c<6.5%)	8 (72.7)	283 (82.3)	248 (82.4)	35 (81.4)
	Diabetic (HbA1c> = 6.5% or on treatment)	3 (27.3)	61 (17.7)	53 (17.6)	8 (18.6)
HbA1c category	Normal HbA1c (<6.5%)	8 (72.7)	291 (84.6)	254 (84.4)	37 (86.0)
	Mild/Mod HbA1c (> = 6.5%-7.9%)	2 (18.2)	24 (7.0)	22 (7.3)	2 (4.7)
	Severe HbA1c (> = 8%)	1 (9.1)	29 (8.4)	25 (8.3)	4 (9.3)
Anaemia Status	Non-Anaemic	4 (36.4)	97 (28.8)	90 (30.7)	7 (15.9)
	Mild Anaemia (Hgb 11.0–12.9/11.0–11.9 g/dL [M/F])	4 (36.4)	94 (27.9)	84 (28.7)	10 (22.7)
	Moderate Anaemia (Hgb 8.0–10.9 g/dL)	1 (9.1)	108 (32.0)	91 (31.1)	17 (38.6)
	Severe Anaemia (Hgb <8 g/dL)	2 (18.2)	38 (11.3)	28 (9.6)	10 (22.7)^b^
HIV Status	Negative	1 (8.3)	116 (33.3)	112 (37.0)	4 (8.9)
	Positive		22 (6.3)	17 (5.6)	5 (11.1)
	Not known (refused)	11 (91.7)	210 (60.3)	174 (57.4)	36 (80.0)
Study Defined TB	Not TB	5 (41.7)	47 (13.5)	40 (13.2)	7 (15.6)
	TB	4 (33.3)	286 (82.2)	252 (83.2)	34 (75.6)
	Unclassifiable	3 (25.0)	15 (4.3)	11 (3.6)	4 (8.9)
Hospital Diagnosed TB	No		34 (10.1)	31 (10.7)	3 (6.7)
	Yes	12 (100.0)	301 (89.9)	259 (89.3)	42 (93.3)
MDR TB	No	4 (100.0)	277 (89.9)	249 (90.5)	28 (84.8)
	Yes (Xpert RIF resistance or on MDR-regimen)		31 (10.1)	26 (9.5)	5 (15.2)
Direct sputum smear microscopy	Acid-fast bacilli negative	4 (33.3)	201 (57.8)	186 (61.4)	15 (33.3)
	Acid-fast bacilli positive		99 (28.4)	84 (27.7)	15 (33.3)
	Missing	8 (66.7)	48 (13.8)	33 (10.9)	15 (33.3)
Albumin (g/L)	Mean (SD)	22.7 (5.8)	23.5 (6.8)	24.2 (6.5)	20.1 (7.2)
Hypoalbuminemia (<20 g/L)	No	4 (66.7)	119 (67.6)	106 (73.6)	13 (40.6)
	Yes	2 (33.3)	57 (32.4)	38 (26.4)	19 (59.4)
C-reactive protein (mmol/L)	Median (IQR)	11.9 (6.5–14.7)	6.9 (2.3–12.2)	6.5 (2.2–11.5)	10.7 (5.2–14.6)
C-reactive protein category	<0.5 mmol/L		19 (6.9)	19 (7.9)	
	0.5–16 mmol/L	7 (77.8)	220 (80.3)	194 (80.2)	26 (81.2)
	>16.0 mmol/L	2 (22.2)	35 (12.8)	29 (12.0)	6 (18.8)
Serum Creatinine (umol/L)	Median (IQR)	69.2 (37.3–86.0)	73.1 (57.1–95.4)	73.1 (57.5–93.5)	73.2 (56.6–108.1)
Serum AST (IU/L)	Median (IQR)	51.0 (33–216)	27.0 (18–47)	27.0 (18–47)	27.0 (18–45)
Serum ALT (IU/L)	Median (IQR)	32.0 (22–53)	27.0 (17–41)	28.0 (17–42)	21.5 (15–31)
Critical AST, ALT, creatinine or blood urea nitrogen^c^	No	3 (50.0)	174 (90.2)	151 (91.0)	23 (85.2)
	Yes	3 (50.0)	19 (9.8)	15 (9.0)	4 (14.8)
Serum Potassium (mmol/L)	Mean (SD)	4.5 (0.9)	4.1 (0.7)	4.2 (0.7)	3.8 (0.8)
Serum Sodium (mmol/L)	Mean (SD)	140.6 (4.8)	138.4 (6.0)	138.9 (5.6)	134.8 (7.9)
Serum Chloride (mmol/L)	Mean (SD)	101.8 (6.1)	100.6 (6.9)	101.3 (6.7)	96.9 (7.0)
Serum Phosphate (mmol/L)	Mean (SD)	1.6 (0.8)	1.3 (0.5)	1.3 (0.5)	1.3 (0.4)
Serum Magnesium (mmol/L)	Mean (SD)	0.8 (0.2)	0.8 (0.1)	0.8 (0.1)	0.8 (0.1)
Serum corrected Calcium (mmol/L)	Mean (SD)	2.4 (0.3)	2.4 (0.2)	2.4 (0.2)	2.4 (0.2)
Any Critical Electrolyte^d^	No	5 (71.4)	214 (80.1)	198 (82.2)	16 (61.5)
	Yes	2 (28.6)	53 (19.9)	43 (17.8)	10 (38.5)^e^
Systemic Inflammatory Response Syndrome (SIRS)^f^	No	1 (8.3)	69 (19.8)	60 (19.8)	9 (20.0)
	Yes	11 (91.7)	279 (80.2)	243 (80.2)	36 (80.0)

BMI, Body Mass Index; MUAC, Mid-Upper Arm Circumference.

^a^ MUAC was significantly lower in the group in whom BMI could not be assessed compared to those with BMI available (t-test, p = 0.0002)

^b^ More severe anaemia was observed in the group without BMI compared to those with BMI data (chi^2^ test for trend, p = 0.003)

^c^ Critical levels defined as 5 x upper limit of normal according to San Lazaro Hospital laboratory reference ranges

^d^ Critical levels of electrolytes were defined according to published criteria [34,39]

^e^ A greater probability of one or more critically low electrolytes was observed in the group without BMI compared to those with BMI data (chi^2^ test p<0.0001)

^f^ SIRS defined as body temperature >38.3° or <36.0°C, heart rate > 90 beats per minute, respiratory rate > 20 breaths per minute, and a total white blood cell count <4 or >12 x 10^9^

Within the primary analysis population, 17.7% (61/344) had newly or previously diagnosed diabetes. The proportion with study defined TB was 82% (286/348), with 53% of those being (151/286) bacteriologically confirmed and 48% (135/282) classified as new cases, 33% as relapse (94/282) and 19% (53/282) as treatment after loss to follow-up or previous treatment outcome unknown. The prevalence of MDR-TB (defined as RIF resistance mutation identified by GeneXpert or already on an MDR treatment regimen) was 10.1% of 308 patients. The proportion of patients who were HIV positive was 6.3% whilst those who were HIV negative was 33%. The remaining had an unknown status due to refusal for HIV screening.

### Inpatient mortality

Inpatient mortality between D3-D28 was 16.7% (58/348; 95% CI 13.1–21.0%). A further 12 deaths occurred during admissions (>28 days), resulting in total inpatient mortality post-D3 was 20.1% (70/348, 95% CI 15.9–24.3%) and 22.8% (82/360, 95% CI 18.4–27.1%) in all enrolled patients (Fig 1).

### Risk factors for inpatient mortality (D3-D28)

**Fig 2:**
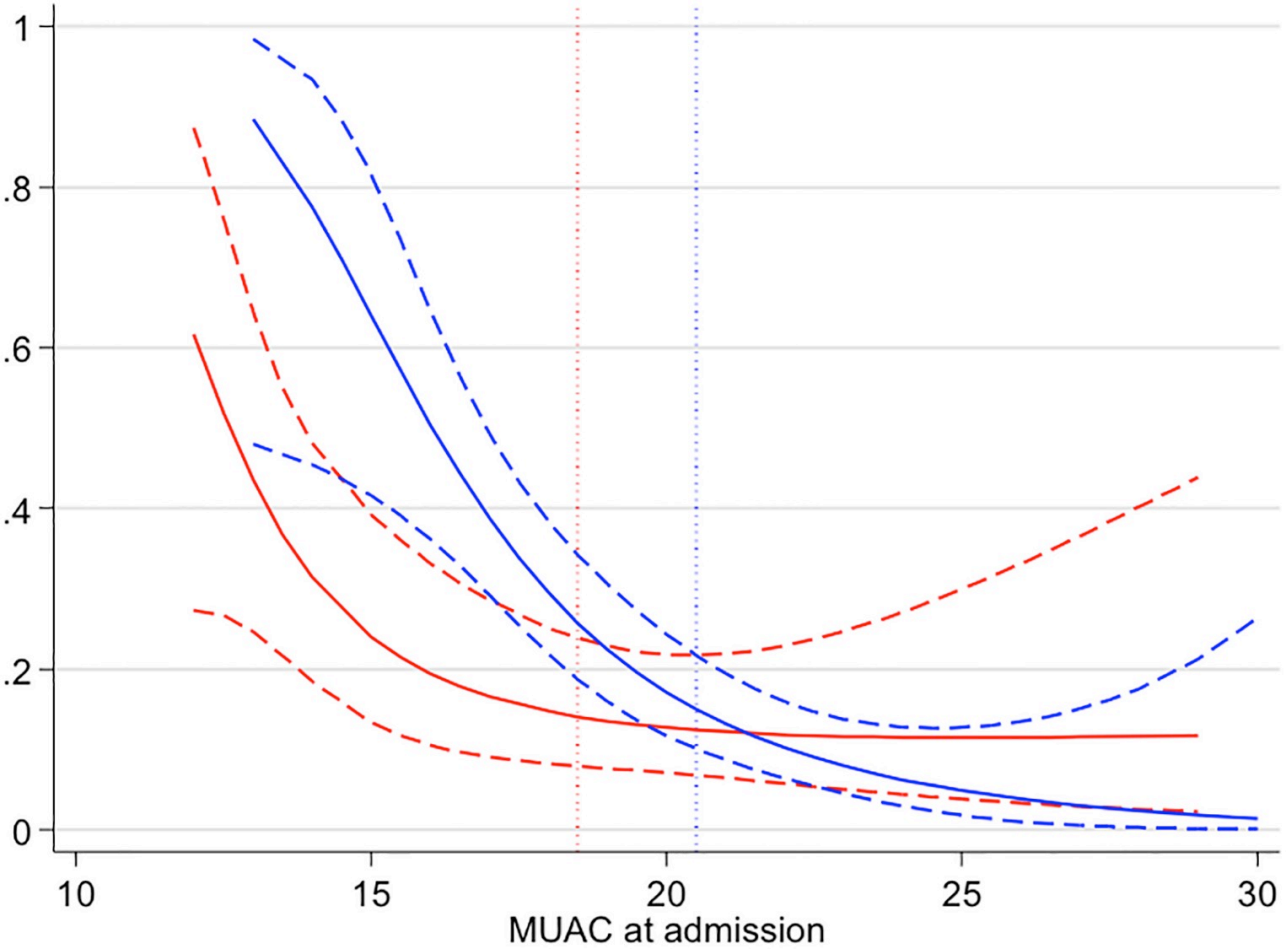
Predicted probability of death by MUAC at admission from unadjusted fractional polynomial logistic regression. Red = females, blue = males, dotted lines are sex specific MUAC cut-off values for under-nutrition.

In univariable analyses, increased odds of in-patient mortality (D3 to D28) were seen for moderate or severe undernutrition assessed by MUAC (p<0.001), MUAC as a continuous variable (Fig 2), missing BMI status compared to BMI > = 17.0 kg/m^2^, lower handgrip strength, lower percentage body fat, both HIV positive status and missing HIV status compared to HIV negative status, and moderate and severe anaemia compared to non-anaemic patients (Table 2). When limited to those with BMI data available, BMI<17 kg/m2 was not associated with in-patient mortality (p = 0.45), whilst MUAC diagnosed moderate/severe under-nutrition was associated, with the effect size increasing in the BMI adjusted model (S2 Table) There was some weak evidence of an association with age (p = 0.070) and for increased risk in those with hospital diagnosed TB (p = 0.051). Higher serum phosphate levels were strongly associated with increased odds of mortality (p = 0.002). Hyperphosphataemia (phosphate >1.6 mmol/L; 38/303) compared to normal or hypophosphataemia (latter defined as phosphate <0.8 mmol/L; 1/303) was associated with increased odds of death (OR = 3.48, 95% CI 1.60–7.60, p = 0.002). Presence of critical electrolyte levels were significantly associated with increased odds of death (p<0.001). A hospital diagnosis of community-acquired pneumonia (CAP) extracted from the discharge/final diagnosis in the clinical notes was also associated with increased odds of mortality (p<0.001). MUAC diagnosed moderate/severe undernutrition was non-significantly associated with increased odds of CAP (OR = 1.44, 95% CI 0.92–2.27; p = 0.112). The presence of systemic inflammatory response syndrome (SIRS) or its individual criteria of tachycardia, tachypnoeia, fever or abnormal white cell count were not associated with mortality. There was no evidence of associations with mortality for level of education or measures of socioeconomic status.

**Table 2:** Univariable associations with D3-D28 mortality (primary analysis population, N = 348).

Characteristic		N	Deaths (%)	OR (95% CI)	p-value
Age (years)	linear effect	348		0.98 (0.96–1.00)	0.070
Sex	Female	106	18 (17.0)	1	0.917
	Male	242	40 (16.5)	0.97 (0.53–1.78)	
BMI Category	> = 17kg/m2	162	16 (9.9)	1	<0.001
	<17kg/m2	141	18 (12.8)	1.34 (0.65–2.73)	
	Not assessable	45	24 (53.3)	10.43 (4.78–22.76)	
MUAC Category	Normal/mild undernutrition	164	13 (7.9)	1	<0.001
	Moderate/severe undernutrition	184	45 (24.5)	3.76 (1.95–7.27)	
Grip strength (kg)	linear effect	346		0.91 (0.88–0.94)	<0.001
% Body Fat	linear effect	335		0.94 (0.90–0.98)	0.001
Diabetic	Non-diabetic (HbA1c<6.5%)	283	48 (17.0)	1	0.450
	Diabetic (HbA1c> = 6.5% or on treatment)	61	8 (13.1)	0.74 (0.33–1.65)	
HbA1c category	Normal HbA1c (<6.5%)	291	48 (16.5)	1	0.426
	Mild/Mod HbA1c (> = 6.5%-7.9%)	24	2 (8.3)	0.46 (0.10–2.02)	
	Severe HbA1c (> = 8%)	29	6 (20.7)	1.32 (0.51–3.42)	
Anaemia Status	Non-Anaemic	97	7 (7.2)	1	0.001
	Mild Anaemia (Hgb 11.0–12.9/11.0–11.9 g/dL [M/F])	94	15 (16.0)	2.44 (0.95–6.29)	
	Moderate Anaemia (Hgb 8.0–10.9 g/dL)	108	21 (19.4)	3.10 (1.26–7.67)	
	Severe Anaemia (Hgb <8 g/dL)	38	14 (36.8)	7.50 (2.72–20.65)	
HIV Status	Negative	116	6 (5.2)	1	<0.001
	Positive	22	6 (27.3)	6.88 (1.98–23.93)	
	Unknown	210	46 (21.9)	5.14 (2.12–12.45)	
Hospital Diagnosed TB	No	34	2 (5.9)	1	0.051
	Yes	301	53 (17.6)	3.42 (0.79–14.71)	
Study Diagnosed TB	No	62	6 (9.7)	1	0.085
	Yes	286	52 (18.2)	2.07 (0.85–5.07)	
Bacteriologically confirmed TB	No	167	19 (11.4)	1	0.072
	Yes	151	28 (18.5)	1.77 (0.94–3.33)	
MDR TB	No	277	39 (14.1)	1	0.761
	Yes (Xpert Rif resist or on MDR-regimen)	31	5 (16.1)	1.17 (0.43–3.24)	
Albumin (g/L)	linear effect	176		0.86 (0.80–0.92)	<0.001
Hypoalbuminemia (<20g/L)	No	119	10 (8.4)	1	<0.001
	Yes	57	24 (42.1)	7.93 (3.44–18.26)	
C-reactive protein (mmol/L)	linear effect	274		1.07 (1.01–1.14)	0.031
C-reactive protein category	<0.5	19	1 (5.3)	1	0.374
	0.5–16	220	34 (15.5)	3.29 (0.43–25.47)	
	>16.0	35	6 (17.1)	3.72 (0.41–33.52)	
Critical AST, ALT, creatinine, or blood urea nitrogen^a^	No	174	30 (17.2)	1	0.056
	Yes	19	7 (36.8)	2.80 (1.02–7.70)	
Serum Potassium (mmol/L)	linear effect	307		0.69 (0.46–1.05)	0.086
Serum Sodium (mmol/L)	linear effect	305		0.94 (0.90–0.99)	0.013
Serum Chloride (mmol/L)	linear effect	305		0.98 (0.93–1.03)	0.408
Serum Phosphate (mmol/L)	linear effect	303		2.60 (1.42–4.76)	0.002
Serum Magnesium (mmol/L)	linear effect	303		5.93 (0.48–72.84)	0.163
Serum corrected Calcium (mmol/L)	linear effect	303		0.72 (0.15–3.32)	0.70
Any Critical Electrolyte^b^	No	214	22 (10.3)	1	0.196
	Yes	53	16 (30.2)	3.77 (1.81–7.86)	<0.001
Clinical diagnosis CAP	No	114	3 (2.6)	1	
	Yes	221	52 (23.5)	11.38 (3.47–37.36)	<0.001

^a^ Critical levels defined as 5 x upper limit of normal according to San Lazaro Hospital laboratory reference ranges

^b^ Critical levels of electrolytes defined according to published criteria [34,39]

After adjustment for age and moderate/severe undernutrition diagnosed by MUAC, odds of death in patients with BMI<17.0 kg/m^2^ were not increased compared to those with BMI≥17.0 kg/m^2^ (OR = 0.38, 95% CI 0.15–0.98), but were substantially increased for patients with missing BMI compared to BMI ≥ 17.0 kg/m^2^ (OR = 2.94, 95% CI 1.07–8.13). It was considered plausible that missing BMI value was a proxy indicator of very poor prognosis based on missing BMI status occurring in immobile and incapacitated patients. A proxy variable for immobility was created based on missing versus non-missing BMI value that was associated with mortality after adjustment for age and under-nutrition based on MUAC (p<0.001).

Increased handgrip strength was associated with decreased odds of mortality after adjustment for age, MUAC and immobility (p<0.001), with the effect of reducing, but not eliminating the effect estimate of MUAC. However handgrip strength was not retained due to MUAC having a much greater potential for practical implementation in this setting. Percentage body fat was not associated with mortality after adjustment for age, MUAC and immobility (p = 0.464).

The final model included age, immobility, anaemia (none or mild, moderate or severe), plus an interaction between sex and MUAC diagnosed moderate or severe undernutrition (LRT p = 0.0316 for interaction) and HIV status (negative, positive or unknown), and clinical diagnosis of CAP (Table 3). Under-nutrition was associated with a large increase in the odds of death in male patients (OR = 5.04, 95% CI 1.50–16.86, p = 0.009). Female patients were more likely to be severely anaemic (20% vs 7%), which was associated with increased odds of death (OR = 9.67 95% CI 2.48–37.76, p<0.001) compared to non-anaemic or mildly anaemic patients. Positive HIV status or missing HIV status (due to lack of consent, often in older patients) and final clinical diagnosis of CAP also had independently increased odds of mortality (Table 3). Due to reduced numbers of observations, hyperphosphataemia was not included in main multivariable analysis, but when included in the same model as reported above (N = 281) was independently associated with increased odds of mortality (OR = 3.74; 95% CI 1.20–11.62; p = 0.023) whilst not appreciably changing the effect estimates of the other independent exposure variables (S3 Table).

**Table 3:** Multivariable analysis of D3-D28 mortality in all admitted patients (N = 325).

Characteristic	Value	Adj OR (95% CI)	p-value
Under-nutrition status in women	Normal/Mild	Ref	
	Moderate/Severe undernutrition	0.81 (0.24–2.77)	0.737
Under-nutrition status in men	Normal/Mild	Ref	
	Moderate/Severe undernutrition	5.04 (1.50–16.86)	0.009
Sex in normal/mild undernutrition	Female	Ref	
	Male	0.38 (0.09–1.58)	0.182
Sex in moderate/severe undernutrition	Female	Ref	
	Male	2.65 (0.63–11.10)	0.182
HIV status	Negative	Ref	
	Positive	6.86 (1.31–35.77)	0.022
	Unknown status	5.46 (1.77–16.84)	0.003
Anemia status	Normal/Mild (Hgb > = 11 g/dL)	Ref	
	Moderate (Hgb 8–10.9 g/dL)	2.58 (0.89–7.45)	0.080
	Severe (Hgb <8 g/dL)	9.67 (2.48–37.76)	<0.001
Immobility	Mobile	Ref	
	Immobile	5.59 (2.25–13.89)	<0.001
Clinical diagnosis CAP	No	Ref	
	Yes	19.23 (4.55–81.20)	<0.001
Age (years)		0.97 (0.94–0.99)	0.030

CAP, Community Acquired Pneumonia

P-values are Wald test p-values. LRT p-value for the interaction term is 0.0316. Overall LRT p-values are: age p = 0.0274; immobility p = 0.002; anaemia p = 0.035; HIV status p = 0.0032; CAP p = <0.0001. Moderate/Severe undernutrition as assessed by MUAC cut-offs of 18.5cm in women and 20.5 cm in men. Immobility assessed as too immobile to get out of bed for measurement of height or weight.

### Diabetes and risk of inpatient mortality

In the univariable analyses, neither diabetes nor level of HbA1C % was associated with inpatient mortality (p = 0.450, p = 0.426; Table 2).

### Subset analyses

When added into the same multivariable model as above, there was no evidence of an association with D3-D28 inpatient death for TB specific factors including: if on anti-tuberculosis treatment (ATT) on admission; new (GeneXpert identified RIF resistance mutation) or existing diagnosis of MDR-TB, basis of TB diagnosis, chest x-ray severity score or presence of cavitation. There was also no association with diabetes or HbA1c levels. Only MTB load from sputum microscopy (semi-quantitative grade) was non-significantly associated. The final multivariable models for study defined TB (N = 283) and bacteriologically confirmed TB cases (N = 151) are shown in Table 4. Evidence of the effect modification of MUAC defined undernutrition by sex remained in the study defined TB subset (LRT p-value for interaction = 0.041) with moderate/severe undernutrition in male TB patients associated with increased odds of mortality but with low precision due to data scarcity (OR = 7.38; 95% CI 1.54–35.38). The interaction term could not be included in the bacteriologically defined sub-set due to reduced observations. Effect estimates for exposure variables included in the final models were similar to those in the main analysis but with reduced precision.

**Table 4:** Multivariable analysis of risk of mortality (D3-D28) in Study-defined TB cases (N = 261) [excluding 3 EXPTB] and Bacteriologically confirmed (N = 140).

		Study Defined TB patients (n = 261)		Bacteriologically confirmed TB (n = 140)	
Characteristic		Adj OR (95% CI)	p-value	Adj OR (95% CI)	p-value
Under-nutrition status in women	Normal/Mild	Ref		-	
	Moderate/Severe undernutrition	0.90 (0.21–3.77)	0.896	-	
Under-nutrition status in men	Normal/Mild	Ref		-	
	Moderate/Severe undernutrition	7.38 (1.54–35.38)	0.012	-	
Under-nutrition in men & women^a^	Normal/Mild	-		Ref	
	Moderate/Severe undernutrition	-		13.21 (1.59–109.61)	0.023
Sex in non-malnourished	Female	Ref		-	
	Male	0.29 (0.05–1.75)	0.177	-	
Sex in malnourished	Female	Ref		-	
	Male	2.35 (0.79–6.98)	0.124	-	
Sex	Female	-		Ref	
	Male	-		1.08 (0.31–3.74)	0.901
HIV status	Negative	Ref		Ref	
	Positive	9.66 (1.54–60.51)	0.015	7.34 (0.78–68.66)	0.081
	Missing	5.04 (1.49–17.02)	0.009	8.09 (1.63–40.23)	0.011
Anemia status	Normal/Mild	Ref		Ref	
	Moderate	2.93 (0.90–9.56)	0.074	2.11 (0.23–19.30)	0.508
	Severe	8.74 (1.84–41.44)	0.006	2.19 (0.17–28.96)	0.553
Mobility	Mobile	Ref		Ref	
	Immobile	5.30 (1.84–41.44)	0.001	11.92 (2.99–47.42)	<0.001
Clinical CAP diagnosis^b^	No	Ref		-	
	Yes	16.16 (3.59–72.79)	<0.001	-	
Age (years)	Linear effect	0.98 (0.95–1.00)	0.095	0.95 (0.91–0.99)	0.033

CAP, Community Acquired Pneumonia

P-values in are Wald test p-values. For the Study Defined TB population LRT p-value for the interaction term is 0.0414. Overall LRT p-values are: age p = 0.0923; immobility p = 0.0010; anaemia p = 0.018; HIV status p = 0.0060; CAP p = <0.0001. Moderate/Severe undernutrition as assessed by MUAC cut-offs of 18.5cm in women and 20.5 cm in men. Immobility assessed as too immobile to get out of bed for measurement of height or weight.

^a^ The MUAC under-nutrition sex interaction could not be modelled in the BC subpopulation due to data sparsity.

^b^ Clinical CAP diagnosis perfectly predicted mortality in the BC subpopulation.

### Mortality post-D28 and post-discharge

There were 12 further inpatient deaths in those that survived until D3. Of the 278 patients discharged alive, attrition was high (Fig 1). Based on known deaths, the minimum mortality by one month after discharge was 4.3% (12/278) in those discharged alive and 23.6% (82/348) in patients alive on D3. By two months, it was 7.2% of those discharged alive and 25.8% in patients alive on D3. By six months the minimum mortality was 9.4% of those discharged alive and 27.6% in patients alive on D3.

### Under-nutrition and all inpatient mortality and total mortality post-D3

Using the same model as above, the effect of MUAC defined moderate or severe undernutrition was also observed for all inpatient mortality after D3 (N = 70 deaths: OR = 4.17 95% CI 1.35–12.91) and to inpatient mortality after D3 and post-discharge mortality combined (N = 96 deaths: OR = 3.35; 95% CI 1.53–7.30) with the effect limited to men in both outcomes and the interaction terms being statistically significant (LRT p-values = 0.0462 and 0.0402).

The primary objective of this prospective cohort study was to compare inpatient mortality of patients with and without evidence of moderate or severe under-nutrition who were admitted to the SLH TB ward. This study demonstrates that the use of sex-specific MUAC cut-offs previously shown in this population to have a high positive predictive value for BMI<17 kg/m^2^ are strongly associated with risk of inpatient death within the first month of admission and excluding early deaths, when nutritional interventions would be unlikely to have an impact [29]. This observation was robust to variations in outcome definition, and patient subsets (all admissions, study defined or bacteriologically confirmed TB). Furthermore, when limited to the subset of those with BMI available, MUAC was associated with risk of death, independent of BMI whilst BMI<17 was not associated. This is very similar to previous observations in emergency admission patients in the UK [40]. Thus MUAC represents a logistically simpler measurement and better predicts risk of death. Moderate evidence suggested a greater effect of under-nutrition on risk of death in males than females. A simple assessment of a patients ability to stand was also strongly and independently associated with risk of death, of a similar effect size as MUAC defined moderate/severe under-nutrition and HIV status. Although body fat percentage and grip strength were also associated with risk of death, MUAC assessment is much simpler and captured more of the variation in outcome. BMI<17 kg/m^2^ has been previously shown in admissions to this ward to be associated with increased risk of inpatient death within 2 weeks in TB-PCR positive admissions (RR 1.95; 95%CI 1.03–3.69), but due to missing data was not reported in multivariable models and no other nutritional assessments were undertaken [15].

We assessed a panel of electrolytes to investigate if these might underlie an effect of acute under-nutrition on inpatient mortality through mechanisms such as dehydration or refeeding syndrome [41]. Similar to previous observations, hyponatraemia was associated with mortality [17]. Contrary to expectations, hyperphosphataemia was associated with mortality, but the aetiology and role of hyperphosphataemia in mortality is unclear.

TB disease and associated mortality are often reportedly higher in males, with epidemiological drivers, delay in seeking treatment, and attrition from treatment programs posited as potential reasons [42]. Increased time-to-treatment is expected to contribute to nutritional deterioration and is often proposed as the underlying reason for observations of low nutritional status being associated with adverse outcomes [6]. However, in this study, there was no evidence of difference in undernutrition by sex in all admissions or in the TB sub-groups. It is possible that sex differences in immune responses extend to TB and is more robust to the effects of under-nutrition in women than men [43].

WHO guidance recommends nutritional assessments for all individuals with active TB, but weights and heights are not measured routinely on admission to the ward and follow-up assessments to track weight gain/loss are not done [26]. In a clinical setting with a high turnover of acutely unwell patients, a simple and quick anthropometric measurement of nutritional state is essential to the diagnosis and management of undernutrition. This is important as despite showing a high prevalence of moderate and severe under-nutrition on the ward it was not recognized or managed by clinical staff. The reasons include limitations in resources (personnel, equipment, interventions, and knowledge), lack of standardized guidelines, the widespread belief that nutritional interventions may be associated with an increased risk of adverse events and that treatment of TB is sufficient for nutritional recovery. Whilst the financial costs of hospital prescribed nutritional support, unlike ATT and basic inpatient costs, have to be borne by the patient. In poor populations with already marginal food intakes and inpatients dependent on standard hospital-provided diets, TB treatment alone is unlikely to be sufficient for nutritional recovery. This is supported by evidence from India in which 23% of patients who completed treatment still had BMI<16 kg/m^2^ from that at diagnosis.^5^ However, surprisingly, there is a very limited evidence base to support the efficacy of interventions to diagnose and treat under-nutrition in TB patients [26,27].

Diabetes was considerably more common in this patient population than in the general population[44]. The lack of association for diabetes or level of HbA1c, indicating the degree of glycaemic dysregulation on risk of death was surprising. However, diabetes was associated with higher BMI (mostly within the normal range) and it is possible that any effects of the diabetes is superseded by the better nutritional status and the advantage that may confer.

A limitation of this study was the unknown HIV status for >50% of participants due to a high testing refusal rate, more common in older patients (100% in > 65 years). However, as older age was not associated with increased risk of death, this does not appear to explain the observation of increased risk of death in those with unknown status. Our study population compared with all admissions to this ward had lower mortality, especially very early, before D3 mortality, but the association between under-nutrition and inpatient death is likely to hold true for all those that survive D3.

## Conclusions

This study establishes the association between under-nutrition assessed using MUAC with risk of inpatient death in this acutely unwell adult population. In this study, there was no apparent effect of diabetes. Further research is required in different populations to optimize MUAC cut-offs to predict risk of death and develop suitable nutritional interventions in active TB disease.

## Supporting information

S1 Table Patterns of missing data. (DOCX)

S2 Table BMI<17 kg/m^2^ and MUAC diagnosed moderate/severe under-nutrition and risk of inpatient mortality (D3-D28) limited to patient subset with BMI data available. Moderate/Severe under-nutrition as assessed by MUAC cut-offs of 18.5cm in women and 20.5 cm in men.
(DOCX)

S3 Table Multivariable analysis of D3-D28 mortality in all admitted patients (N = 281) including hyperphosphataemia. P-values are Wald test p-values. LRT p-value for the interaction term is 0.0257. Overall LRT p-values are: age p = 0.033; immobility p = 0.0087; anaemia p = 0.0149; HIV status p = 0.0053; CAP p = <0.0001; hyperphosphataemia p = 0.024. Moderate/Severe undernutrition as assessed by MUAC cut-offs of 18.5cm in women and 20.5 cm in men. Immobility assessed as too immobile to get out of bed for measurement of height or weight.
(DOCX)
