# Supplementary material for: Mid-upper arm circumference predicts death in adult patients admitted to a TB ward in the Philippines: A prospective cohort study
Source: PLoS One. 2019 Jun 27;14(6):e0218193. doi: 10.1371/journal.pone.0218193 (PMC6597043; doi:10.1371/journal.pone.0218193)
Supplement: S1 Table — (DOCX) [file pone.0218193.s001.docx]

**S2 Table. Patterns of Missing Data**

|  | **Died <D3 N=12** | **Analysis set N=348** | **Analysis set with BMI N=303** | **Analysis set with missing BMI N=45** |
| --- | --- | --- | --- | --- |
| **Characteristic** | **% missing** | % missing | **% missing** | **% missing** |
| Food Intake Past 24hr | 66.7 | 34.5 | 30.4 | 62.2 |
| HbA1c Result | 8.3 | 1.1 | 0.7 | 4.4 |
| Diabetes | 8.3 | 1.1 | 0.7 | 4.4 |
| Anaemia Status | 8.3 | 3.2 | 3.3 | 2.2 |
| Hospital Diagnosed TB | 0 | 3.7 | 4.3 | 0 |
| MDR TB status | 66.7 | 11.5 | 9.2 | 26.7 |
| Albumin | 50.0 | 49.4 | 52.5 | 28.9 |
| Serum Creatinine (umol/L) | 50.0 | 21.0 | 21.1 | 20.0 |
| CRP (mmol/L) | 25.0 | 21.3 | 20.1 | 28.9 |
| Serum AST (IU/L) | 41.7 | 29.9 | 31.0 | 22.2 |
| Serum ALT (IU/L) | 25.0 | 23.0 | 22.8 | 24.4 |
| Critical Liver or Kidney Lab | 50.0 | 44.5 | 45.2 | 40.0 |
| Potassium (mmol/L) | 16.7 | 11.8 | 11.2 | 15.6 |
| Sodium (mmol/L) | 16.7 | 12.4 | 11.6 | 17.8 |
| Chloride (mmol/L) | 33.3 | 41.1 | 42.6 | 31.1 |
| Inorganic Phosphate mmol/L | 41.7 | 12.9 | 10.6 | 28.9 |
| Magnesium (mmol/L) | 41.7 | 12.9 | 10.6 | 28.9 |
| Calcium (mmol/L) | 41.7 | 12.9 | 10.6 | 28.9 |
| Critical Electrolyte Lab | 41.7 | 23.6 | 20.5 | 42.2 |
| Critical White Cell value | 8.3 | 3.2 | 3.3 | 2.2 |
