# Supplementary material for: Mid-upper arm circumference predicts death in adult patients admitted to a TB ward in the Philippines: A prospective cohort study
Source: PLoS One. 2019 Jun 27;14(6):e0218193. doi: 10.1371/journal.pone.0218193 (PMC6597043; doi:10.1371/journal.pone.0218193)
Supplement: S2 Table — Moderate/Severe under-nutrition as assessed by MUAC cut-offs of 18.5cm in women and 20.5 cm in men. (DOCX) [file pone.0218193.s002.docx]

**S2 Table. BMI<17 kg/m^2^ and MUAC diagnosed moderate/severe under-nutrition and risk of inpatient mortality (D3-D28) limited to patient subset with BMI data available (N=303).**

| **Characteristic** | **Value** | **OR (95% CI)** | **p-value** |
| --- | --- | --- | --- |
| BMI kg/m^2^ | Normal/Mild [BMI>17] | Ref |  |
|  | Mod/Severe under-nutrition [BMI ≤17 kg/m^2^] | 1.36 (0.65-2.73) | 0.428 |
| MUAC | Normal/Mild | Ref |  |
|  | Mod/Severe under-nutrition | 2.37 (1.11-5.06] | 0.025 |
|  |  | **Adj OR (95% CI)** |  |
| BMI kg/m^2^ | Normal/Mild [BMI>17] | Ref |  |
|  | Mod/Severe under-nutrition [BMI ≤17 kg/m^2^] | 0.50 (0.18-1.36) | 0.175 |
| MUAC | Normal/Mild | Ref |  |
|  | Mod/Severe under-nutrition | 3.95 (1.38-11.32) | 0.010 |
