# Supplementary material for: Mid-upper arm circumference predicts death in adult patients admitted to a TB ward in the Philippines: A prospective cohort study
Source: PLoS One. 2019 Jun 27;14(6):e0218193. doi: 10.1371/journal.pone.0218193 (PMC6597043; doi:10.1371/journal.pone.0218193)
Supplement: S3 Table — P-values are Wald test p-values. LRT p-value for the interaction term is 0.0257. Overall LRT p-values are: age p = 0.033; immobility p = 0.0087; anaemia p = 0.0149; HIV status p = 0.0053; CAP p = <0.0001; hyperphosphataemia p = 0.024. Moderate/Severe undernutrition as assessed by MUAC cut-offs of 18.5cm in women and 20.5 cm in men. Immobility assessed as too immobile to get out of bed for measurement of height or weight. (DOCX) [file pone.0218193.s003.docx]

**S3 Table. Multivariable analysis of D3-D28 mortality in all admitted patients (N=281) including hyperphosphataemia**

| **Characteristic** | **Value** | **Adj OR (95% CI)** | **p-value** |
| --- | --- | --- | --- |
| Malnutrition status in women | Normal/Mild | Ref |  |
|  | Moderate/Severe undernutrition | 0.59 (0.12-2.87) | 0.513 |
| Malnutrition status in men | Normal/Mild | Ref |  |
|  | Moderate/Severe undernutrition | 5.87 (1.36-25.44) | 0.018 |
| Sex in normal/mild undernutrition | Female | Ref |  |
|  | Male | 0.32 (0.06-1.67) | 0.177 |
| Sex in moderate/severe undernutrition | Female | Ref |  |
|  | Male | 3.20 (0.90-11.36) | 0.072 |
| HIV status | Negative | Ref |  |
|  | Positive | 5.12 (0.70-37.63) | 0.109 |
|  | Unknown status | 6.44 (1.87-22.17) | 0.003 |
| Anemia status | Normal/Mild (Hgb >= 11 g/dL) | Ref |  |
|  | Moderate (Hgb 8-10.9 g/dL) | 2.46 (0.70-8.66) | 0.159 |
|  | Severe (Hgb <8 g/dL | 8.70 (1.87-40.37) | 0.006 |
| Immobility | Mobile | Ref |  |
|  | Immobile | 4.51 (1.47-13.85) | 0.009 |
| Clinical diagnosis CAP | No | Ref |  |
|  | Yes | 27.65 (4.85-157.74) | <0.001 |
| Hyper hosphataemia | No (serum phosphate <=1.6mmol/L) | Ref |  |
|  | Yes (serum phosphate >1.6mmol/L) | 3.74 (1.20-11.62) | 0.023 |
| Age (years) |  | 0.96 (0.93-0.99) | 0.037 |
